# Massive hemoptysis in mycobacterium abscessus lung disease: Interventional management with bronchial artery embolization

**DOI:** 10.1016/j.idcr.2026.e02548

**Published:** 2026-03-11

**Authors:** Poonam Patil, Babaji Ghewade, Ulhas Jadhav, Amit Toshniwal, Amisha Soni

**Affiliations:** Department of Respiratory Medicine, Datta Meghe Institute of Higher Education and Research (DMIHER), Deemed University, Sawangi (Meghe), Wardha, India

**Keywords:** Bronchiectasis, Mycobacterium abscessus, Non-tuberculous Mycobacteria, Hemoptysis, Bronchial Artery Embolization, Lady Windermere Syndrome

## Abstract

In older women without traditional risk factors, Lady Windermere syndrome is a recognized phenotypic presentation of non-tuberculous mycobacterial (NTM) pulmonary disease that is traditionally linked to Mycobacterium avium complex (MAC) infection. It is characterized by a nodular-bronchiectatic pattern that usually affects the lingula and right middle lobe. Hemoptysis is a known sequence of NTM, severity of which may vary from minor episode to multiple, fatal. We present a case of a female treated case of tuberculosis and then presenting

with massive hemoptysis. Radiological assessment showed bronchiectasis and nodular infiltrates in the right middle lobe consistent with Lady Windermere syndrome. Sputum examination confirmed the growth of mycobacterium abcessus. Hemoptysis caused by this infective agent was massive and non-refractory to medical treatment. Bleeding from several feeder vessels was promptly controlled by an urgent bronchial artery embolization. Patient was started on targeted, evidence based treatment regimen for mycobacterium abcessus and had reserved lung functions, maintained sputum conversion and was symptomatically better at an 18- months follow-up. This case highlights the association of non-mycobacterium with hemoptysis severe enough to require bronchial artery embolization. For best results, it emphasizes on the necessity for a multidisciplinary strategy that combines the management of an infectious illness with radiological intervention.

## Introduction

Lady Windermere syndrome is an unique nodular-bronchiectatic condition of the lung caused by spectrum of non- tuberculous- mycobacterium, first described by Reich and Johnson [1]. It has been associated with behavioral pattern of subconscious cough suppression in elderly women [1], [2]. It is believed that NTM colonization and subsequent infection are facilitated by decreased mucociliary clearance in the right middle lobe and lingula, which are commonly affected by the syndrome. Although the most commonly implicated pathogen is Mycobacterium avium complex (MAC), other NTM species, such as the more aggressive and challenging-to-treat Mycobacterium abscessus complex, can exhibit the same clinical and radiological presentation [3].

One frequent and potentially dangerous side effect of NTM pulmonary illness is hemoptysis. Studies have shown that a significant percentage of patients experience it, leading to a growing recognition of its prevalence. About 18% of patients with NTM lung disease needed bronchial artery embolization (BAE) to control hemoptysis, which was seen in 42.6% of the patients in a large Korean cohort [4]. In this case, hemoptysis is caused by a variety of causes, including bronchiectasis, chronic inflammation, and related necrotizing granulomas that erode hypertrophied, fragile bronchial arteries. Massive hemoptysis is a life-threatening emergency that needs to be treated right once. It is commonly characterized as more than 200–300 milliliters in a 24-hour period. With immediate control rates above 90%, bronchial artery embolization (BAE) has emerged as the preferred non-surgical first-line treatment for large and recurring hemoptysis [5], [6].

We present a case of massive hemoptysis in a patient with Lady Windermere syndrome caused by M. abscessus, successfully managed with emergency BAE followed by targeted medical therapy. This report aims to provide a detailed account of the clinical presentation, discuss the critical role of BAE in the context of NTM-related hemoptysis, and review the relevant literature on this therapeutic approach.

## Case presentation

In her fifties, a thin built woman arrived at the emergency room with acute-onset severe hemoptysis. She gave the history that over the 24 h before admission, she coughed up about 300 and 400 milliliters of fresh bright red colored blood along with significant anxiety and worsening dyspnea corresponding to mMRC grade III. She had pulmonary tuberculosis that had been treated eight years prior, and there were no known after effects of chronic lung illness. She had no substantial occupational exposures and no history of smoking.

On examination, the patient was tachypneic with blood pressure of 130/70 mm of Hg, pulse rate of 120 beats per minute, respiration rate of 32 cycles per minute, and her oxygen saturation was 92% on room air. Respiratory system evaluation revealed coarse crackles confined to the bottom and middle zones of the right lung. Signs of immunodeficiency, connective tissue disease, and heart failure were not present.

Initial laboratory investigations revealed a hemoglobin of 10.4 g/dL, leukocytosis of 11,000 cells/µL, and a markedly elevated C-reactive protein of 70 mg/L. Renal function tests and liver function tests were within normal limits. Given the history of prior TB and the current presentation, serial sputum examinations was done. Two sputum samples, collected one week apart, were positive for acid-fast bacilli (1 +) on Ziehl-Neelsen staining. However, GeneXpert MTB/RIF assays on both samples were negative for Mycobacterium tuberculosis complex. This discordance prompted strong clinical suspicion for NTM disease, and samples were sent for mycobacterial culture.

**Fig. 1:**
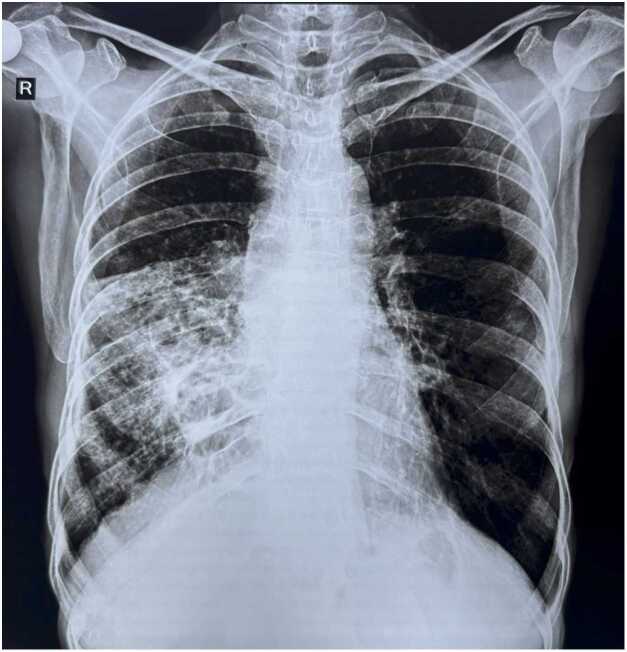
Chest X-ray posteroanterior (PA) view suggestive of right middle and lower zone heterogenous opacity.

**Fig. 2:**
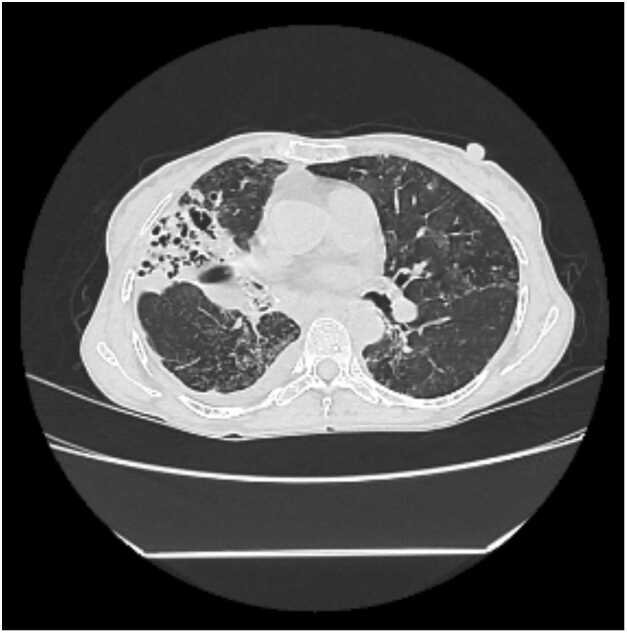
High-resolution contrast-enhanced computed tomography (CECT) of the thorax.

A chest radiograph (Fig. 1) revealed heterogeneous opacity in the right middle and lower zones. A contrast-enhanced high-resolution computed tomography (CECT) of the thorax was performed urgently to delineate the extent of parenchymal disease and localize the source of bleeding. The scan (Fig. 2) demonstrated severe cystic and cylindrical bronchiectasis with surrounding tree-in-bud nodularity, confined almost exclusively to the right middle lobe and lingula, a pattern classic for Lady Windermere syndrome. No Rasmussen's aneurysm, active cavitary lesion, or aspergilloma was identified.

**Fig. 3:**
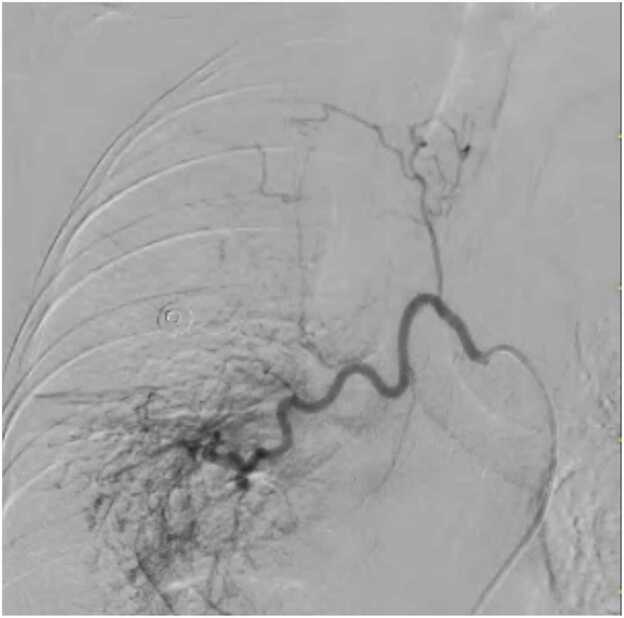
Bronchial Angiography Pre-embolization angiogram showing a hypertrophied bronchial artery trunk with multiple abnormal, tortuous feeder vessels (arrowheads) supplying the right middle lobe.

The patient was admitted to the intensive care unit, and administered intravenous tranexamic acid. Despite this, she experienced another episode of massive hemoptysis. An emergency bronchial angiogram was performed, which revealed hypertrophied and tortuous bronchial arteries with multiple feeder vessels arising from the right intercostobrachial trunk, supplying the bronchiectatic right middle lobe. These were successfully and selectively embolized using 355–500 µm polyvinyl alcohol (PVA) particles (Fig. 3). Post-embolization angiography confirmed complete cessation of blood flow to the abnormal vessels. The patient experienced immediate and complete cessation of hemoptysis.

Following the procedure, the patient's condition stabilized. Several weeks later, the sputum culture results confirmed the growth of Mycobacterium abscessus. Susceptibility testing indicated inducible macrolide resistance. A multidisciplinary team discussion led to the initiation of a guideline-based multidrug regimen, consisting of oral azithromycin 500 mg daily, oral linezolid 600 mg twice daily, and oral clofazimine 100 mg twice daily. She was discharged with close outpatient follow-up.

**Fig. 4:**
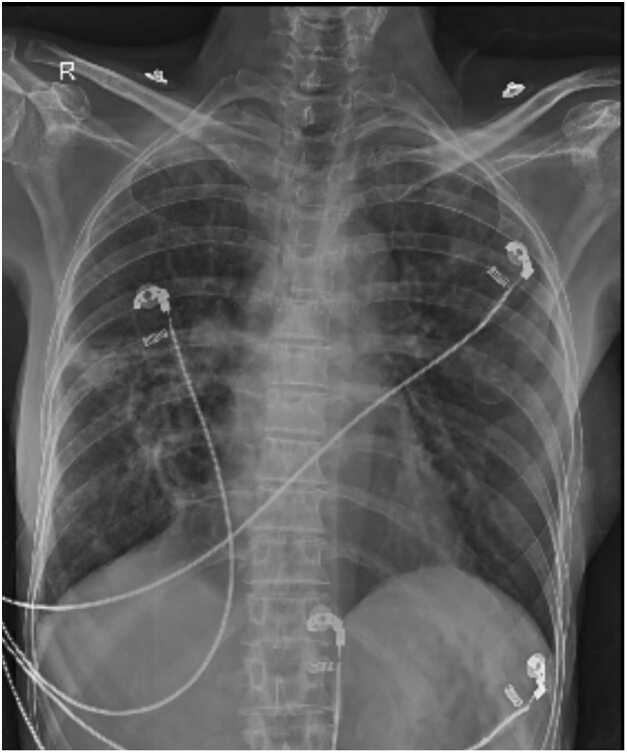
Follow-up Chest X-ray (PA view). Image obtained 18 months post-treatment shows marked resolution of the previous right middle and lower zone opacity, with residual fibrotic changes.

The patient demonstrated excellent adherence to therapy. Serial sputum cultures were performed every two months and converted to negative after nine months of treatment. She completed a full 12 months of therapy after the date of the first negative culture (total 21 months of treatment). At her 18-month follow-up, she remained asymptomatic, with no recurrence of hemoptysis, and a repeat chest radiograph showed significant resolution of the right middle lobe opacities (Fig. 4). Her lung function remained stable.

Axial image demonstrating severe cystic and cylindrical bronchiectasis with extensive tree-in-bud nodularity in the right middle lobe, characteristic of nodular-bronchiectatic NTM disease (Lady Windermere syndrome).

## Discussion

This case illustrates the successful management of a life-threatening complication of NTM pulmonary disease. It is valuable for several reasons: it highlights

(1) an atypical pathogen, M. abscessus, presenting as classic Lady Windermere syndrome;
(2) the severity of hemoptysis that can occur in this condition; and
(3) the central, life-saving role of bronchial artery embolization (BAE) as a bridge to definitive medical therapy.

While Lady Windermere syndrome is most often linked to MAC, our case adds to the growing body of evidence that other NTM species, particularly the M. abscessus complex, can produce an identical clinical phenotype [3], [7]. This is a crucial distinction, as the management of M. abscessus is significantly more challenging due to its intrinsic multidrug resistance, requiring prolonged multidrug therapy with a high likelihood of recurrence [8]. The patient's prior history of tuberculosis likely contributed by causing structural damage, creating a nidus that predisposed to subsequent NTM colonization and infection.

### Hemoptysis in NTM disease: a significant clinical challenge

Hemoptysis is a common and often underappreciated complication of NTM pulmonary disease. Although the stated incidence varies, its clinical relevance is confirmed by vast series. In a retrospective review of 787 patients with NTM lung disease, Lee et al. discovered that 335 (42.6%) of them had hemoptysis at some point in their illness [4]. Importantly, BAE was needed for control in 61 patients, or 18.2% of those with hemoptysis. The amount of hemoptysis that requires BAE might vary from little but frequent to large and potentially fatal. Chronic inflammation that causes neo-vascularization, hypertrophy, and tortuosity of the systemic bronchial arteries is the underlying pathogenesis. These delicate arteries are located next to bronchiectatic airways and are vulnerable to rupture, particularly during coughing fits or infection flare-ups. This consequence seems to be more common in the nodular-bronchiectatic type of the disease, as observed in our case [4].

### Bronchial artery embolization: technique and outcomes in NTM

The main interventional technique for treating hemoptysis of different causes is now BAE. It has a well-established involvement in hemoptysis related to NTM. After selective catheterization of the bronchial arteries, which are the cause of bleeding in more than 90% of cases, coils or particles (such PVA or gelatin sponge) are used for embolization. Reducing arterial pressure at the bleeding site is the main objective in order to enable hemostasis [5], [6].

In the majority of series, including those that concentrate on NTM patients, the immediate clinical success rate of BAE for hemoptysis management is routinely reported above 85–95% [4], [6], [9]. BAE was incredibly successful in stopping the patient's severe bleeding right away. Nevertheless, BAE is a temporary solution that manages the bleeding without addressing the underlying inflammatory and infectious disease. Consequently, hemoptysis recurrence is a known danger, especially during the first six to twelve months, and is frequently linked to infection progression or relapse [10]. Pre-procedural fungal balls, cavities, and multibed artery embolization were all recognized by Li et al. as risk factors for recurrence [10]. Lee et al. found that 31% of NTM patients who had BAE experienced recurrence, underscoring the urgent necessity for efficient post-embolization antibiotic treatment to deal with the underlying etiology [4]. The fact that our patient is still in remission at 18 months shows how effective it is to combine timely BAE with focused, extended antibiotic therapy.

### Comprehensive management of *M. abscessus* Lady Windermere Syndrome

As was the case in this instance, the care of *M. abscessus* lung illness is infamously challenging and necessitates a protracted, multimodal approach based on susceptibility testing and the presence or absence of inducible macrolide resistance [8]. According to current guidelines, the initial intense phase should involve both oral and intravenous antibiotics, with a continuation phase involving oral medications [8]. Based on susceptibility patterns and practicality for long-term outpatient care, an oral regimen of azithromycin, linezolid, and clofazimine was selected for our patient. A favorable outcome is indicated by sputum conversion at nine months and persistent negativity following treatment completion. In order to effectively manage both the acute emergency and the persistent infection, this case highlights the need for a multidisciplinary strategy comprising pulmonologists, interventional radiologists, and infectious disease specialists.

## Conclusion

According to this case report, Mycobacterium abscessus may be the cause of Lady Windermere syndrome, a disorder that is typically linked to MAC and manifests as extensive hemoptysis. It emphasizes how crucial bronchial artery embolization is as a very successful, life-saving treatment for managing severe hemoptysis in this situation. The report highlights that BAE is an essential link to definitive therapy rather than a stand-alone treatment. Prompt, focused, and sustained antimicrobial treatment against the particular NTM pathogen is necessary for a favorable long-term outcome. Clinicians should maintain a high index of suspicion for NTM disease in patients with bronchiectasis and non-resolving symptoms, be aware of the risk of hemoptysis, and be prepared to utilize interventional radiology techniques as part of a comprehensive management strategy.

## CRediT authorship contribution statement

**Babaji Ghewade:** Supervision. **Ulhas Jadhav:** Validation. **Poonam Patil:** Writing – original draft, Investigation, Formal analysis, Conceptualization. **Amit Toshniwal:** Data curation. **Amisha Soni:** Writing – review & editing.

## Author contributorship statements

Poonam Patil: Contributed to writing - original draft, editing, data curation.

Babaji Ghewade and Ulhas Jadhav: Contributed to resources, supervision, formal analysis, validation, review.

Amit Toshniwal, Amisha Soni: Contributed to data curation, review, editing.

## Ethics statement

Written informed consent was obtained from the patient for publishing this case along with accompanying images in biomedical journals for research purposes.

## Funding

This report received no specific grant from any funding agency in the public, commercial, or not-for-profit sectors.

## Declaration of Competing Interest

All the authors declare no conflicts of interest related to this case report. No financial or personal relationships could have influenced the work reported in this paper.
